# Full Genome Analysis of Influenza A(H1N1)pdm09 Virus Isolated from Peru, 2013

**DOI:** 10.1128/genomeA.00191-14

**Published:** 2014-04-17

**Authors:** Carlos Padilla, Fredy Condori, Maribel Huaringa, Pool Marcos, Nancy Rojas, Victoria Gutierrez, Omar Cáceres

**Affiliations:** a Biotechnology and Molecular Biology Laboratory, Instituto Nacional de Salud, Lima, Peru; b National Reference Laboratory of Respiratory Virus (NIC-191), Instituto Nacional de Salud, Lima, Peru

## Abstract

The pandemic influenza A(H1N1)pdm09 virus has been reported in Peru since 2009. We report the whole-genome sequence analysis of a viral isolate from an infection case that occurred during an influenza outbreak in 2013. This strain shows novel hemagglutinin (HA) mutations that may cause an antigenic drift that diminishes the protective effect of the vaccine.

## GENOME ANNOUNCEMENT

The pandemic influenza A(H1N1)pdm09 virus was first reported in Peru in May 2009. During the first months of the epidemic, school-age children and young adults were affected (1). Later, the disease caused by this virus extended around the country, affecting other groups. Between May and December 2009, this epidemic caused 8,994 laboratory-confirmed cases (2). The pandemic influenza cases during 2010 to 2012 declined and were replaced by cases caused by the A(H3N2) viral subtype (F. Condori, unpublished data). However, from July to August 2013, another influenza outbreak occurred in Peru, during which 5,757 samples were analyzed and 1,105 cases were confirmed to be caused by A(H1N1)pdm09 by real-time PCR in the NIC-191 of the National Institute of Health of Peru.

In order to evaluate the genomic changes in isolates of A(H1N1)pdm09 during 2013, we sequenced the whole genome of a strain isolated from a patient with a mild infection. All eight influenza gene segments were amplified in an overlapping manner by one-step real-time reverse transcription-PCR (RT-PCR) using the whole-genome primers recommended by the WHO and CDC (3). Sequencing was carried out using the BigDye Terminator version 3.1 cycle sequencing kit (ABI), and processing for capillary electrophoresis was carried out on an ABI 3500XL DNA analyzer. The sequences obtained were assembled using the SeqScape software, and the sequence alignment of each gene was carried out using MEGA version 5.2.

All gene segments of the Peruvian strain were compared with those of the vaccine component strain A/California/07/2009 and those of the currently circulating strains. The total genome has 13,160 bp, with 43.33% G+C content. The results showed nucleotide and amino acid changes in all segments of the genome. The hemagglutinin (HA) gene shows 97.88% amino acid identity with that in the vaccine strain, and the mutations K180Q (in the antigenic site Sa) and E391K were identified. This E391K mutation has been associated with cases of mild infection (4). Other mutations present in the HA gene included P100S, D114N, S202T, S220T, A273T, K300E, I338V, S468N, and E516K. This strain remains sensitive to neuraminidase (NA) inhibitor drugs, like oseltamivir; however, it presented new mutations: L40V, N44S, N200S, and N248D. The mutations V241I and N369K, which are associated with stability of the resistant virus (5), were also detected in this strain. No mutations were found to be associated with amantadine resistance on the membrane (M) gene, but the mutations V80I, M192V, and K230R were present. Similarly, the mutations for the polymerase basic 2 (PB2) gene were R54K, M66I, D195M, R293K, V344M, I354L, and V731I; for PB1, the mutations were S98T, G154D, Y397M, and I435T; for the polymerase acidic (PA) gene, they were P224S, N321K, and A343T; for the nonstructural (NS) gene, they were L90I, I123V, and N205S; and for the nucleoprotein (NP) gene, they were V100I and S498N. The presence of the S220T (HA), N248D (NA), I123V (NS), and V100I (NP) mutations placed the Peruvian strain in clade 7 (6).

One of the main concerns derived from our analysis is the possibility that identified and forthcoming novel HA mutations may cause an antigenic drift that would be sufficient to diminish the protective effect of vaccination against A(H1N1)pdm09. This concern seems relevant because the viral strain utilized for vaccine development (the influenza A/California/7/2009 strain) does not carry the mutant form of the HA protein (5).

### Nucleotide sequence accession numbers.

The whole-genome sequence of the Peruvian A(H1N1)pdm09 isolate from 2013 has been deposited in DDBJ/ENA/GenBank under accession no. KJ147484 to KJ147491.
